# Clinical Profile and Prognosis of a Real-World Cohort of Patients With Moderate or Severe Cancer Therapy-Induced Cardiac Dysfunction

**DOI:** 10.3389/fcvm.2021.721080

**Published:** 2021-10-29

**Authors:** Alberto Esteban-Fernández, Juan Fernando Carvajal Estupiñan, Juan José Gavira-Gómez, Sonia Pernas, Pedro Moliner, Alberto Garay, Álvaro Sánchez-González, Inmaculada Fernández-Rozas, José González-Costello

**Affiliations:** ^1^Cardiology Service, Hospital Universitario Severo Ochoa, Leganés, Spain; ^2^Cardiology Service, Instituto del Corazón de Bucaramanga, Bucaramanga, Colombia; ^3^Cardiology Department, Clínica Universidad de Navarra, Pamplona, Spain; ^4^Oncology Department, Hospital Duran i Reynals, Institut Català d'Oncologia, L'Institut d'Investigació Biomèdica de Bellvitge (IDIBELL), L'Hospitalet de Llobregat, Barcelona, Spain; ^5^Cardiology Department, Hospital Universitari de Bellvitge, IDIBELL, L'Hospitalet de Llobregat, Barcelona, Spain; ^6^Bladder, Functional and Oncological Pathology Unit, Urology Department, Hospital Universitario Ramón y Cajal, Instituto Ramón y Cajal de Investigación Sanitaria (IRYCIS), University of Alcalá, Madrid, Spain

**Keywords:** cardio-oncology, cancer therapy-related cardiac dysfunction, cardiotoxicity, left ventricular systolic dysfunction (LVSD), cancer therapies

## Abstract

**Introduction and Objectives:** Cancer therapy-related cardiac dysfunction (CTRCD) is a common cause of cancer treatment withdrawal, related to the poor outcomes. The cardiac-specific treatment could recover the left ventricular ejection fraction (LVEF). We analyzed the clinical profile and prognosis of patients with CTRCD in a real-world scenario.

**Methods:** A retrospective study that include all the cancer patients diagnosed with CTRCD, defined as LVEF < 50%. We analyzed the cardiac and oncologic treatments, the predictors of mortality and LVEF recovery, hospital admission, and the causes of mortality (cardiovascular (CV), non-CV, and cancer-related).

**Results:** We included 113 patients (82.3% women, age 49.2 ± 12.1 years). Breast cancer (72.6%) and anthracyclines (72.6%) were the most frequent cancer and treatment. Meantime to CTRCD was 8 months, with mean LVEF of 39.4 ± 9.2%. At diagnosis, 27.4% of the patients were asymptomatic. Cardiac-specific treatment was started in 66.4% of patients, with LVEF recovery-rate of 54.8%. Higher LVEF at the time of CTRCD, shorter time from cancer treatment to diagnosis of CTRCD, and younger age were the predictors of LVEF recovery. The hospitalization rate was 20.4% (8.8% linked to heart failure). Treatment with trastuzumab and lower LVEF at diagnosis of CTRCD were the predictors of mortality. Thirty point nine percent of patients died during the 26 months follow-up. The non-CV causes and cancer-related were more frequent than CV ones.

**Conclusions:** Cardiac-specific treatment achieves LVEF recovery in more than half of the patients. LVEF at the diagnosis of CTRCD, age, and time from the cancer treatment initiation to CTRCD were the predictors of LVEF recovery. The CV-related deaths were less frequent than the non-CV ones. Trastuzumab treatment and LVEF at the time of CTRCD were the predictors of mortality.

## Key Points

What is known about the topic?

In the patients with CTRCD, the cardiac-specific treatment can lead to LVEF recovery, although there is a lack of evidence about the clinical profile and the best strategy to manage these patients. Moreover, cardiotoxicity leads to the cancer treatment withdrawal, which impacts prognosis.

What does it bring back?

Cardio-oncology units can provide an early diagnosis and treatment of cardiotoxicity. The multidisciplinary teams can also allow continuing the cancer treatments, improving prognosis in the patients with CTRCD, emphasizing that most deaths are due to non-CV ones, such as cancer.

## Introduction

A cancer therapy-related cardiac dysfunction (CTRCD) is a structural or functional myocardial injury secondary to cancer treatment. The cardiac damage depends on the type, dosage, and schedule of cancer therapies and other factors, such as the pre-existing cardiovascular (CV) risk factors and cardiac disease, age, or prior exposure to cardiotoxic therapy. In addition, cancer itself has been related to the increased cardiac peptides, associating higher mortality from any cause (1).

The incidence of CTRCD varies according to the definition and series. In a recently published multicenter registry, the incidence of cardiotoxicity, defined as a decrease in left ventricular ejection fraction (LVEF) below 50%, elevated cardiac biomarkers, or presence of other abnormal echo parameters, such as a significant decrease in the global longitudinal strain (GLS), reached 37.5%. The advanced stages of CTRCD imply left ventricular systolic dysfunction (LVSD), with different ranges of ejection fraction impairment (2). However, there is a lack of clinical trial-based evidence on the specific management of patients with LVSD secondary to the cancer treatment, and they are treated similarly to the rest of patients with LVSD (3). Recently, sacubitril-valsartan showed a potential benefit in cardiac remodeling in oncological patients (4, 5).

The prognosis of the patients with CTRCD seems to be worse than in other cardiomyopathies (6), and in the patients with heart failure (HF), non-CV causes of death, especially cancer, were more prevalent than HF progression or sudden death (7). Additionally, the cardiotoxic effects are a critical treatment-limiting adverse effect of some chemotherapies and targeted therapies, affecting the prognosis (8). It is essential to implement the multidisciplinary cardio-oncology units to diagnose and treat LVSD early (3).

Although the possibility of cardiotoxicity due to the cancer treatment is well-known, there is a lack of studies describing the clinical profile, LVEF dynamics, and the prognosis of patients with CTRCD in a real-world setting. Our study aimed to analyze the clinical profile, management, and prognosis of a cohort of oncological patients with moderate or severe cardiotoxicity in a real-world clinical practice.

## Materials and Methods

### Study Population and Ethics

We retrospectively included all the consecutive patients diagnosed with CTRCD in the two tertiary hospitals in Spain: Clínica Universidad de Navarra between 2000 and 2011 and Hospital Universitari de Bellvitge between 2010 and 2016. The patients with cancer were referred to the cardiology units by the oncologists or hematologists to evaluate and treat cardiotoxicity before implementing the cardio-oncology units in these centers. The LVSD was defined as LVEF < 50%, with a previously normal value, after cancer treatment administration, such as chemotherapy and targeted therapies, such as immunotherapy. The patients with baseline cardiac function not available or those with a previous history of LVSD were excluded. The study complied with the Declaration of Helsinki, and all the patients gave consent for using their data for research purposes.

### Baseline Assessment and Follow-Up

The baseline characteristics, cardiovascular risk factors, type of cancer, and treatment received were recorded. The baseline cardiac function was analyzed by either echocardiogram or single photon emission computed tomography (SPECT) before the treatment and monitored periodically during follow-up to detect CTRCD. Both the methods have been validated to assess the myocardial function in the patients with cancer (9, 10). We analyzed the cardiac-specific treatment prescribed after the LVSD diagnosis according to medical criteria and recommendations of the European Society for Medical Oncology (ESMO) (11) and European Society of Cardiology (ESC) HF guidelines (2), defined as starting treatment with at least an angiotensin-converting enzyme inhibitors (ACE-I) or an angiotensin receptor blockers (ARB). The follow-up was performed during the routine clinic visits by reviewing the electronic medical records. We recorded different events: the New York Heart Association (NYHA) functional class, hospital admission, medical treatment changes, LVEF recovery, the need for heart transplantation, and death (CV, cancer, and non-CV). The heart transplantation was considered a CV death in the survival analysis. The LVEF recovery was defined as LVEF ≥ 50% at any time during the follow-up.

### Statistical Analysis

The quantitative variables are expressed as mean and SD or median and interquartile range (IQR) when data did not fit a normal distribution and the qualitative variables as number and percentage. The continuous quantitative variables were compared using the Student's *t*-test or the sum of Wilcoxon's ranges for non-normally distributed variables. The categorical variables were compared with the chi-square test and Fisher's exact test when appropriate. A significance level of ≤ 0.05 (bilateral) was established for all the statistical tests.

The survival distribution related to time to an event was evaluated using the Kaplan–Meier method, in the whole population, and according to LVEF recovery. The log-rank test was employed to compare the survival curves.

A multivariate binary logistic regression analysis was performed to evaluate the LVEF recovery predictors, using the sequential inclusion and exclusion method, with inclusion threshold *p* < 0.05 and exclusion higher than 0.2 in the univariate analysis. A multivariate Cox proportional hazards regression model was conducted to calculate the adjusted hazard ratios (HR) and to determine the effect of several variables on survival function. A univariate analysis was performed to select the variables for both the multivariate analyses, with inclusion threshold *p* < 0.05 and exclusion >0.2. In the multivariate analysis, a *p* < 0.05 was considered statistically significant. Age was selected for the multivariate analysis due to its clinical relevance. A statistical analysis was performed with SPSS 23.0 (IBM, NY, USA).

## Results

We included 113 patients whose baseline characteristics are summarized in Table 1. The most frequent diagnoses were breast cancer (72.6%) and hematological malignancies (15.9%). In total, 72% of the patients underwent surgery, and 53.1% thoracic radiotherapy. The most used chemotherapy treatments were: anthracyclines (72.6%), cyclophosphamide (47.8%), and trastuzumab (33.6%).

**Table 1:** The baseline characteristics of patients with cancer therapy-related cardiac dysfunction (CTRCD).

**Baseline characteristics**	**All patients** **(*n =* 113)**	**LVEF recovery** **(*n =* 63)**	**Non-LVEF recovery** **(*n =* 21)**	**Unknown LVEF recovery** **(*n =* 29)**	*p*-value^*^
Sex (female)-*n* (%)	93 (82.3)	54 (85.7)	17 (81.0)	7 (24.1)	0.508
Age (years old)	49.2 (12.1)	49.6 (11.4)	51.1 (13.3)	47.0 (12.9)	0.620
Tobacco history-*n* (%)					0.756
*Never smoker*	85 (75.2)	49 (77.7)	15 (71.4)	21 (72.4)	
*Past smoker*	15 (13.3)	6 (9.5)	4 (19.0)	5 (17.2)	
*Current smoker*	13 (11.5)	8 (12.7)	2 (9.5)	3 (10.3)	
Arterial hypertension-*n* (%)	21 (18.6)	15 (23.8)	6 (28.6)	0 (0)	0.011
Diabetes-*n* (%)	6 (5.3)	4 (6.4)	2 (9.5)	0 (0)	0.325
Dyslipidemia-*n* (%)	15 (13.3)	9 (14.3)	4 (19.0)	2 (6.9)	0.519
BMI (kg/m^2^)	25.4 (3.8)	25.4 (4.1)	24.9 (3.5)	26.6 (1.3)	0.777
Previous cancer-*n* (%)	15 (13.3)	9 (14.3)	2 (9.5)	4 (13.8)	0.868
Chronic kidney disease-*n* (%)	0 (0)				
Previous cardiopathy-*n* (%)	3 (2.7)	2 (3.2)	1 (4.8)	0 (0)	0.594
Baseline LVEF (%)	57.7 (5.5)	58.4 (5.8)	56.7 (4.5)	56.9 (5.6)	0.210
Previous HF-*n* (%)	0 (0)				
SBP (mmHg)	119.1 (14.2)	117.6 (13.3)	124.2 (16.6)	124.7 (21.4)	0.292
Previous AF-*n* (%)	2 (1.8)	2 (3.2)	0 (0)	0 (0)	0.833
HR (bpm)	84.0 (9.6)	84.2 (9.7)	82.3 (7.7)	85.7 (15.3)	0.667
Prior medical treatment-*n* (%)					0.186
*Beta-blockers*	1 (0.9)	0 (0)	1 (4.8)	(0)	
*ACE-I/ARB*	11 (9.7)	9 (14.3)	2 (9.5)	(0)	
*Digoxin*	1 (0.9)	1 (1.6)	0 (0)	(0)	
*Diuretics*	6 (5.3)	5 (7.9)	1 (4.8)	(0)	
*Statins*	6 (5.3)	3 (4.8)	1 (4.8)	2 (6.9)	
Type of cancer-n (%)					0.880
*Breast cancer*	82 (72.6)	49 (77.8)	15 (71.4)	18 (62.1)	
*Hematological diseases*	18 (15.9)	9 (14.3)	3 (14.3)	6 (20.7)	
*Bone cancer*	4 (3.5)	2 (3.2)	1 (4.8)	1 (3.5)	
*Gynecologic non-breast cancer*	3 (2.7)	1 (1.6)	1 (4.8)	1 (3.5)	
*Other*	6 (5.3)	2 (3.2)	1 (4.8)	3 (10.3)	
Chemotherapy agent-*n* (%)					
*Trastuzumab*	38 (33.6)	32 (50.8)	3 (14.3)	3 (10.3)	0.004
*Anthracycline*	82 (72.6)	46 (73.0)	16 (76.2)	20 (69.0)	0.750
*Cyclophosphamide*	54 (47.8)	33 (52.4)	8 (38.1)	11 (37.9)	0.236
*Docetaxel*	36 (31.9)	23 (36.5)	4 (19.0)	9 (31.0)	0.130
*Cisplatin/Carboplatin*	24 (21.2)	17 (27.0)	3 (14.3)	4 (13.8)	0.560
*Gemcitabine*	10 (8.8)	2 (3.2)	4 (19.0)	4 (13.8)	0.015
*Fluorouracil*	35 (31.0)	19 (30.2)	5 (23.8)	11 (37.9)	0.556
*Paclitaxel*	25 (2.1)	14 (22.2)	6 (28.6)	5 (17.2)	0.584
Thoracic radiotherapy-*n* (%)	60 (53.1)	37 (58.7)	8 (38.1)	15 (51.7)	0.396
Time from chemotherapy to CTRCD (months)					
*Median time*	8 (4, 19)	9 (4, 17)	5 (2.5; 12)	5 (2.5; 12)	0.221
*Mean time*	30.2 (57.8)	27.8 (54.3)	65.4 (86.2)	9.8 (12.8)	0.07
Start cardiac- specific treatment-*n* (%)	75 (66.4)	54 (85.7)	17 (81)	4 (13.8)	0.127
Death patients-*n* (%)	35 (30.9)	20 (31.7)	7 (33.3)	8 (27.6)	0.297
*CV causes*	8 (7.1)	3 (4.8)	4 (19.1)	1 (3.5)	0.06
*Non-CV causes*	12 (10.6)	8 (12.7)	1 (4.8)	3 (10.3)	0.552
*Cancer*	15 (13.3)	9 (14.3)	2 (9.5)	4 (13.8)	0.804

*ACE-I, angiotensin-converting-enzyme inhibitor; AF, atrial fibrillation; ARB, angiotensin II receptor blockers; BMI, body mass index; BPM, beats per minute; CTRCD, cancer therapy-related cardiac dysfunction; CV, cardiovascular; HF, heart failure; HR, heart rate; LVEF, left ventricular ejection fraction; MRA, mineralocorticoid receptor antagonist; SBP, systolic blood pressure*.

*Cardiac-specific treatment meant to receive at least ACE-I/ARB after CTRCD diagnosis*.

* *p-value is calculated to compare LVEF recovery and non-LVEF recovery patients*.

Cancer therapy-related cardiac dysfunction was diagnosed by echocardiography in 68 patients (60.2%) and SPECT in 45 (39.8%). The mean LVEF was 39.4 ± 9.2% at the time of diagnosis of CTRCD (Table 2). The median time from starting chemotherapy to the diagnosis of cardiotoxicity was 8 months [IQR: 4–19]. At the time of diagnosis, 31 patients (27.4%) were asymptomatic, 41 were in NYHA II (36.3%), 13 in NYHA III (11.5%), and 5 in NYHA IV (4.4%). Of the symptomatic ones, 25 (21.2%) had been admitted for HF. In 43 patients (38.1%), the diagnosis of CTRCD implied a change in the chemotherapy dose-schedule or withdrawal of cancer therapy.

**Table 2:** Evolution of left ventricular ejection fraction (LVEF) during follow-up of patients with cancer therapy-related to cardiomyopathy.

**Parameter**	**Baseline**	**Diagnosis** **of CTRCD**	**Last** **Follow-up**	***p*-value**
LVEDD (mm)	49.5 (4.4)	54.7 (5.2)	49.1 (5.5)	0.0001
LVESD (mm)	35.7 (4.1)	42.7 (6.1)	34.3 (5.8)	0.0001
LVEDV (mL)	118.8 (22.1)	140.5 (34.6)	107.3 (33.0)	0.002
LVESV (mL)	55.3 (12.9)	83.7 (25.8)	46.1 (19.5)	0.0001
LVEF (%)	58.2 (5.5)	38.8 (8.5)	54.1 (9.2)	0.0001
LVEF in recovered patients (%)	58.3 (5.8)	39.6 (8.3)	56.8 (5.6)	0.0001
LVEF in patients with persistence of LVSD (%)	57.7 (2.9)	34.1 (8.9)	38.3 (9.9)	0.0001

*LVEDD, end-diastolic diameter of the left ventricle; LVEDV, end-diastolic volume of the left ventricle; LVESD, end-systolic diameter of the left ventricle; LVESV, end-systolic volume of the left ventricle; LVEF, left ventricular ejection fraction; LVSD, left ventricular systolic dysfunction*.

*The baseline values are those before starting the cancer treatment. LVEDD, LVEDV, LVESD, and LVESV are only presented when we had echocardiographic studies (n = 68)*.

**Figure 1:**
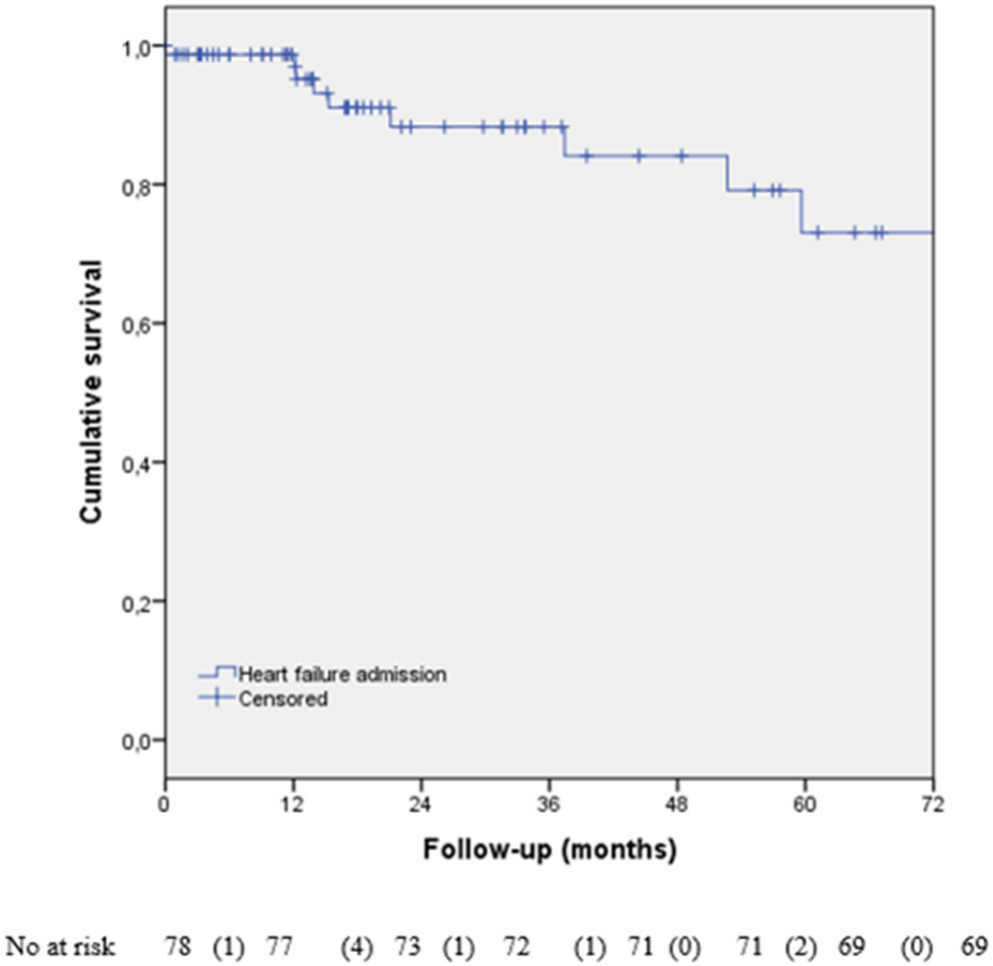
Kaplan–Meier analysis of heart failure (HF) hospital admission during follow-up. We have lost data about HF admission in the 35 patients.

The cardiac-specific treatment was started in 75 patients (66.4%), as shown in Table 3. After a median follow-up of 26.2 months [IQR:12.2–94.5], most of the patients were in NYHA I (39.5%) or II (48.1%). Twenty-three patients were admitted to the hospital (one for a CV event, 10 for HF (Figure 1), and 12 for non-CV causes, excluding cancer routine admissions).

**Table 3:** Treatment initiated in the patients with cancer therapy-related cardiomyopathy.

**Drug**	**Total** **(*n =* 75)**	**Patients with** **LVEF recovery** **(*n =* 54)**	**Patients with** **LSVD persistence** **(*n =* 17)**	***p*-value**
Beta-blocker-*n* (%)	56 (74.7)	38 (70.4)	14 (82.4)	0.888
*Carvedilol*	45 (60.0)	33 (61.1)	9 (52.9)	0.029
*Bisoprolol*	10 (13.3)	5 (9.3)	5 (29.4)	
*Nevibolol*	1 (1.3)	1 (1.9)	0 (0)	
ACE-I-*n* (%)	62 (82.7)	44 (81.5)	14 (82.4)	0.174
*Enalapril*	52 (69.3)	37 (68.5)	11 (64.7)	0.545
*Ramipril*	7 (9.3)	5 (9.3)	2 (11.8)	
*Otros*	2 (2.7)	1 (1.9)	1 (5.9)	
ARB-*n* (%)	11 (14.6)	8 (14.8)	3 (17.6)	0.770
*Losartan*	5 (6.6)	5 (9.3)	0 (0)	0.048
*Valsartan*	4 (5.3)	3 (5.6)	1 (5.9)	
*Candesartan*	2 (2.7)	0 (0)	2 (11.8)	
MRA-*n* (%)	15 (20.0)	10 (18.5)	3 (17.6)	0.663
*Spironolacton*	12 (16.0)	9 (16.7)	2 (11.8)	0.242
*Eplerenone*	3 (4.0)	1 (1.9)	1 (5.9)	
Digoxin-*n* (%)	8 (10.7)	5 (9.3)	3 (17.6)	0.816
Diuretics-*n* (%)	44 (58.7)	27 (50.0)	14 (82.4)	0.209

*ACE-I, angiotensin-converting-enzyme inhibitor; ARB, angiotensin II receptor blockers; LVEF, left ventricular ejection fraction; LVSD, left ventricular systolic dysfunction; MRA, mineralocorticoid receptor antagonists*.

*We excluded four patients from the sub-analysis due to lost data regarding LVEF recovery*.

Left ventricular ejection fraction was recovered in 62 patients (54.8%), 54 of them (87.1%) receiving cardiac-specific treatment. LVSD persisted in 21 patients (18.6%). LVEF determination during the follow-up was lost in 30 patients (26.5%), but there were data about survival status in all the patients included. After LVEF recovery, 37 patients (58.7%) continued cardiac-specific treatment, as is shown in Table 4. Among the patients who continued treatment, eight patients presented recurrent LVEF dysfunction, five of them due to the new chemotherapy treatment.

**Table 4:** Maintenance of cardiac treatment in the patients with LVEF recovery after cancer therapy-related cardiomyopathy.

**Drug**	**Patients** **(*n =* 37)**	***p*-value**	**Time (months)**
Beta-blockers-*n* (%)	37 (100)		12 [IQR: 7 to 33]
*Carvedilol*	31 (83.8)	0.039	
*Bisoprolol*	6 (16.2)		
ACE-I-*n* (%)	35 (94.6)		13 [IQR: 6.8 to 34.5]
*Enalapril*	33 (89.2)	0.24	
*Ramipril*	2 (5.4)		
ARB-*n* (%)	9 (24.3)		24 [IQR: 24 to 24]
*Valsartan*	4 (10.8)	0.13	
*Losartán*	5 (13.5)		
ARM-*n* (%)	13 (35.1)		
*Spironolacton*	12 (32.4)	0.009	
*Eplerenone*	1 (2.7)		
Digoxin-*n* (%)	4 (10.8)		14.5 [IQR: 8.8 to 21.8]
Diuretics-*n* (%)	16 (43.2)		
Statins-*n* (%)	16 (43.2)		

*ACE-I, angiotensin-converting-enzyme inhibitor; ARB, angiotensin II receptor blockers; ARM, mineralocorticoid receptor antagonist*.

*Time refers to the median time that the cardiac-specific treatment was maintained after LVEF recovery*.

In the binary logistic regression analysis (Table 5), higher LVEF at the time of CTRCD [*OR* 1.13; *CI* 95% 1.03–1.25; *p* = 0.008], shorter time from starting chemotherapy to the diagnosis of CTRCD [*OR* 0.99; *CI* 95% 0.98–1.00; *p* = 0.023], and younger age [*OR* 0.94; *CI* 95% 0.88–0.99; *p* = 0.03] were identified as the predictors of LVEF recovery, independently of trastuzumab treatment, HF admission at diagnosis of CTRCD, and Carvedilol treatment after dysfunction. For each 5% of LVEF increase at the time of diagnosis of CTRCD, the probability of recovery of LVEF increased by 1.75.

**Table 5:** Binary logistic regression analysis to identify the predictors of LVEF recovery in the patients with cancer therapy-related cardiomyopathy.

**Predictors of LVEF recovery**						
	**Univariate**			**Multivariate**		
	**OR**	**CI 95%**	***p*** **-value**	**OR**	**CI 95%**	***p*** **-value**
Age (1 year)	0.99	0.95–1.03	**0.63**	0.94	0.88–0.99	**0.03**
Female sex	1.39	0.38–5.08	0.62			
No smoking history	0.93	0.18–4.98	0.94			
Arterial hypertension	0.80	0.26–2.42	0.79			
Dyslipidemia	0.72	0.20–2.64	0.62			
Diabetes	0.66	0.11–3.86	0.64			
BMI (1 kg/m^2^)	1.03	0.85–1.24	0.77			
Baseline LVEF (1%)	1.07	0.97–1.17	0.20			
LVEF at the time of diagnosis of CTRCD (1%)	1.10	1.03–1.2	**0.002**	1.13	1.03–1.25	**0.008**
Trastuzumab treatment	6.00	1.60–22.46	**0.008**	3.15	0.44–22.8	0.26
Anthracyclines treatment	0.83	0.26–2.61	0.75			
Thoracic radiotherapy	2.1	0.75–6.03	0.16			
HF admission at CTRCD diagnosis	0.35	0.10–1.28	0.11			
Cardiac specific treatment	1.56	0.42–5.80	0.51			
Beta-blocker treatment	0.80	0.28–2.29	0.68			
Carvedilol treatment	4.58	1.02–20.69	**0.048**	1.78	0.27–11.6	0.55
ACE-I treatment	1.19	0.41–3.45	0.74			
ARB treatment	0.91	0.22–3.78	0.89			
MRA treatment	1.04	0.25–4.26	0.96			
Time from starting chemotherapy to dysfunction (1 month)	0.99	0.98–1.00	**0.035**	0.99	0.98–1.00	**0.023**

*ACE-I, angiotensin-converting-enzyme inhibitor; ARB, angiotensin II receptor blockers; BMI, body mass index; CI, confidence interval; CTRCD, cancer therapy-related cardiac dysfunction; CV, cardiovascular; HF, heart failure; LVEF, left ventricular ejection fraction; MRA, mineralocorticoid receptor antagonist; OR, odds ratio*.

*Cardiac-specific treatment meant to receive at least ACE-I/ARB after CTRCD diagnosis. Bold means statistically significant (p < 0.05)*.

In addition, we identified the treatment with trastuzumab [*HR* 1.25; *CI* 95% 1.02–4.96; *p* = 0.045] and lower LVEF at the time of diagnosis of CTRCD [*HR* 0.94; *CI* 95% 0.91–0.97; *p* = 0.0001] as the predictors of mortality independent of age, dyslipidemia, anthracyclines treatment, and LVEF recovery during the follow-up (Table 6).

**Table 6:** The Cox regression analysis to identify the predictors of mortality in the patients with cancer therapy-related cardiomyopathy.

**Predictors of mortality**						
	**Univariate**			**Multivariate**		
	**HR**	**CI 95%**	***p*** **-value**	**HR**	**CI 95%**	***p*** **-value**
Age (1 year)	1.01	0.98–1.03	**0.69**	1.01	0.98–1.05	0.482
Female sex	1.24	0.54–2.84	0.62			
No smoking history	1.03	0.42–0.25	0.96			
Arterial hypertension	1.00	0.35–2.88	0.99			
Dyslipidemia	5.58	0.76–40.78	**0.09**	5.73	0.78–42.0	0.09
Diabetes	1.12	0.15–8.32	0.92			
BMI (1 kg/m^2^)	0.88	0.63–1.22	0.44			
Baseline LVEF (1%)	0.97	0.90–1.03	0.31			
LVEF at the time of diagnosis of CTRCD (1%)	0.94	0.91–0.97	**0.001**	0.94	0.91–0.97	**0.0001**
Trastuzumab treatment	1.83	0.84–3.98	**0.14**	2.25	1.02–4.96	**0.045**
Anthracyclines treatment	1.88	0.95–3.70	**0.07**	1.56	0.78–3.12	0.212
Thoracic radiotherapy	1.37	0.70–2.67	0.35			
HF admission at diagnosis	1.64	0.77–3.51	0.20			
Cardiac specific treatment	0.75	0.29–1.99	0.56			
Beta-blocker treatment	0.79	0.38–1.63	0.52			
Carvedilol treatment	0.43	0.05–3.49	0.43			
ACE-I treatment	1.58	0.70–3.60	0.27			
ARB treatment	0.21	0.03–1.55	0.13			
MRA treatment	1.95	0.83–4.59	0.13			
Time from starting chemotherapy to dysfunction (1 month)	1.00	0.99–1.01	0.72			
LVEF recovery during follow-up	1.33	0.54–1.33	0.53			
HF admission during follow-up	1.09	0.32–3.73	0.89			

*ACE-I, angiotensin-converting-enzyme inhibitor; ARB, angiotensin II receptor blockers; BMI, body mass index; CI, confidence interval; CTRCD, cancer therapy-related cardiac dysfunction; CV, cardiovascular; HF, heart failure; HR, hazard ratio; LVEF, left ventricular ejection fraction; MRA, mineralocorticoid receptor antagonist*.

*Cardiac-specific treatment meant to receive at least ACE-I/ARB after CTRCD diagnosis. Bold means statistically significant (p <0.05)*.

**Figure 2:**
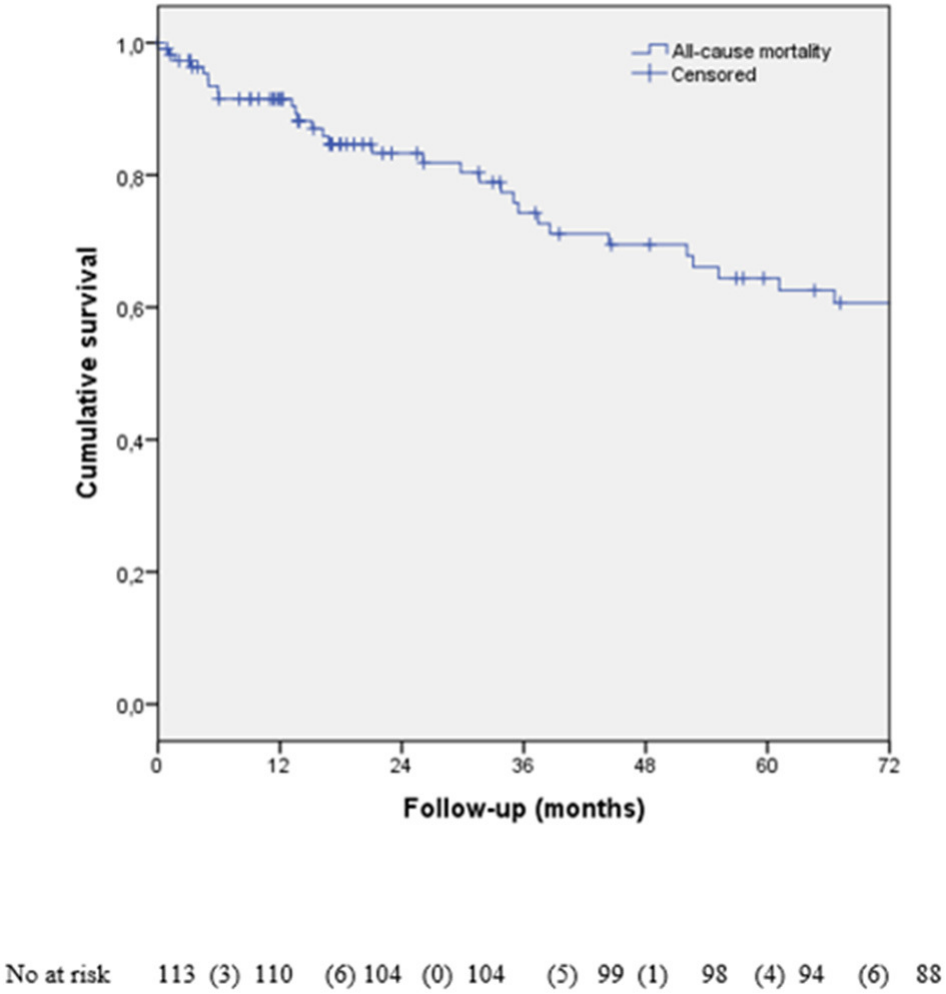
Kaplan–Meier survival analysis according to all-cause of death.

**Figure 3:**
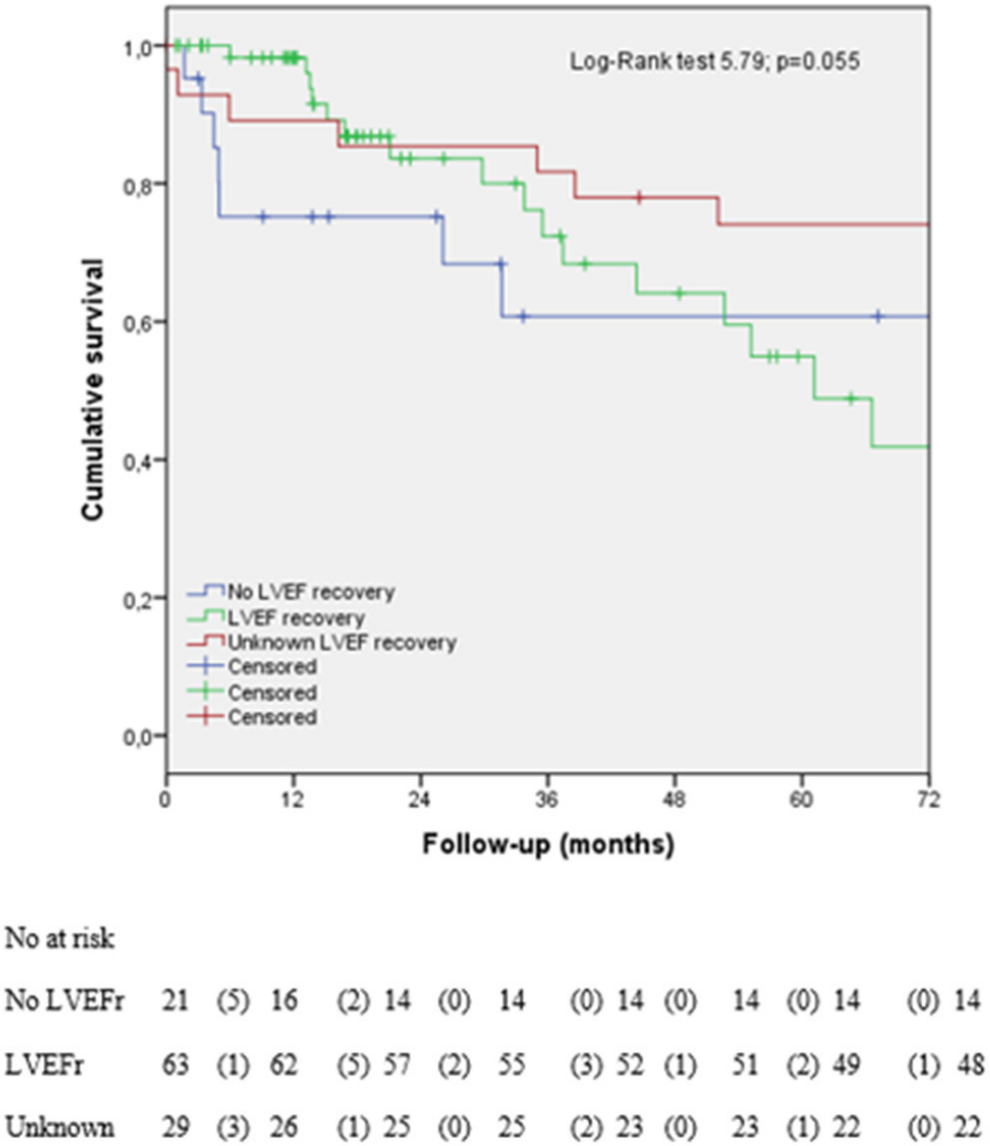
Kaplan–Meier survival analysis according to left ventricular ejection fraction (LVEF) recovery. LVEF, left ventricular ejection fraction; LVEFr, left ventricular ejection fraction recovery. Unknown refers to the patients with lost data about LVEF recovery. Heart transplantation was included as cardiovascular (CV) death.

As it is shown in Figure 2, 78 (69.1%) patients were alive, and 35 (30.9 %) had died at the end of follow-up (six of CV causes and two were transplanted, 15 of cancer and 12 of non-CV causes) (Table 1). There were no differences in the mortality according to the presence of LVEF recovery, but there was a trend to earlier mortality from the CV causes in those with the absence of LVEF recovery (Figure 3).

## Discussion

This is one of the largest real-world cohorts reported in the literature of patients with moderate to severe CTRCD and long-term follow-up to the best of our knowledge. The main findings of our study were: (1) with appropriate cardiac treatment in 66% of all patients, up to 55% of patients achieve LVEF recovery; (2) early CTRCD diagnosis is associated with improved LVEF recovery after initiation of cardiac-specific treatment; (3) less advanced LVSD at the time of CTRCD diagnosis is associated with the improved LVEF recovery and increased overall survival, and (4) all-cause mortality in the patients with CTRCD was ~40% at 5 years of follow-up. There was a trend to earlier mortality from the CV causes in those that did not achieve LV recovery. Our findings emphasize the need to develop multidisciplinary cardio-oncology units to make an early diagnosis of CTRCD and start the cardiac-specific treatment as soon as possible to improve the prognosis.

Some factors associated with CTRCD are CV risk factors, older age, or ischemic disease. However, in our real-life cohort, most of them were young women with breast cancer and low incidence of CV risk factors. In other registers, breast cancer and hematological diseases were the most frequent ones (12). The criteria for CTRCD diagnosis varied in different studies, and we established a cut-off of LVEF <50% according to the ESC guidelines and other similar studies (2, 7). All the patients in our cohort had reduced to mid-range ejection fraction (LVEF 39.4 ± 9.2%).

Depending on the type of cancer, treatment schedule, and individual characteristics, some studies reported that the cardiotoxicity usually appeared during the first year after starting chemotherapy (13–15), similar to our cohort (8 months). Most importantly, LVEF at the moment of diagnosis of CTRCD was linked to LVEF recovery and mortality in our study, emphasizing the need to perform early diagnosis of CTRCD and initiate the treatment before LVEF deteriorates further that include new parameters, such as GLS. This is important as 27.4% of our patients were asymptomatic at the time of diagnosis. Thus, the development of protocols with the periodic cardiac function assessment is necessary to detect CTRCD, as the time to dysfunction after starting the cancer drug treatment was one of the most relevant parameters for LVEF recovery in our study.

Early medical treatment has been demonstrated to improve LVEF (13), and the ESC guidelines recommended the cardiac-specific treatment in the symptomatic patients with LVSD (2). In the patients with CTRCD, the evidence with ACE-I and beta-blockers in the asymptomatic patients was limited to the SAVE trial in ischemic patients (16) and SAFEHEART to prevent the development of symptoms (17). When our study was performed, the international guidelines recommended that the patients who developed CTRCD during or following treatment with Type II agents (i.e., trastuzumab) in the absence of anthracyclines could be observed if they remained asymptomatic and LVEF remained ≥40% (11). This explains why 34% of patients in our series did not receive cardiac-specific treatment, as many of our patients had mid-range LVEF and were asymptomatic. In these cases, trastuzumab was interrupted, and LVEF was reassessed 1 month later without cardiac-specific treatment initiation. This could have led to slower or less LVEF recovery and more interruptions of treatment with trastuzumab, which could have influenced the fact that trastuzumab was associated with increased mortality in our series. Also, it may be possible that the increase of mortality with trastuzumab happened in more advanced oncological patients.

In our study, 87.1% of patients who recovered LVEF received the cardiac-specific treatment, emphasizing the need for cardio-oncology units to start the cardiac treatment as early as possible in all the patients with CTRCD to improve the outcomes. The most employed drugs were ACE-I and beta-blockers, specially Carvedilol and Enalapril, similar to other studies (18). Martin-Garcia et al. recently demonstrated in a small 67 patients study that sacubitril-valsartan improved the remodeling and functional status in the patients with CTRCD (5), so future studies should focus on this possibility.

Left ventricular ejection fraction recovery rate varied according to the definition in different studies. Cardinale et al. distinguished between the partial recovery (LVEF increase >5 total points and >50%) and full recovery (LVEF recovery to baseline) (13). Lupon et al., in a study with 1,057 patients with HF and LVSD, considered recovered a LVEF ≥ 45% after a previous one of <45% (19). In the study of Pareek et al., with 535 patients with CTRCD, they reported a recovery rate of 94% with the treatment, but they considered a LVEF change from 45 to 53% (20). In our study, we established a cut-off of 50% to consider LVEF recovery. In total, 55% of our cohort recovered LVEF, similar to other studies [Cardinale et al. [42%] (18), Hamirani et al. [44%] (15), or Ohtani et al. [67.3%] (21)].

The presence of severe CTRCD (18) and the use of drugs, such as anthracyclines (22) have been related to non-reversible myocardial damage. However, although most of the patients received anthracyclines in our cohort, more than half of them recovered LVEF, probably because of the cardiac-specific treatment and a short time to CTRCD diagnosis.

The mean follow-up of our cohort was similar to CARDIOTOX (12), but some of our patients, especially with LVSD persistence, had long-term follow-up (26 months, IQR 12.2–94.5). Hospital admission during CTRCD has been poorly studied. Yoon et al. reported 9% of HF admission and 11% of symptomatic patients during the follow-up (23). In another study, only 10% of patients were in NYHA III-IV during follow-up, similar to our cohort (12.4%) (20). The hospital admission rate of our cohort was 20.4%, mainly due to non-CV causes (excluding cancer routine admission), with only 8.8% because of HF, similar to Yoon et al. study (23).

The long-term maintenance of cardiac-specific treatment after recovery was supported by treatment for heart failure in patients with recovered dilated cardiomyopathy (TRED-HF) trial (24). In our study, 58.7% of the patients maintained long-term cardiac treatment after recovery. In the study by Pareek et al., 88% of the patients continued cancer treatment after cardiac optimization and close follow-up in a cardio-oncology unit (20), slightly above what was achieved in our cohort (62%). If the patients needed to continue the cancer treatments, we should avoid cardiotoxic ones to reduce the risk of further new dysfunction, as 12.9% of our patients with LVEF recovery had recurrent LVSD in our study. Some studies demonstrated that LVEF recovery improved the morbidity and mortality (19), but it remained controversial in the patients with CTRCD. Yoon et al., in a study with 243 patients, reported the worst outcomes in the non-recovered group (symptomatic HF, HF hospitalization, and death), but they did not analyze the mortality separately (23). Our study did not see the statistical differences in mortality in the patients with LVEF recovery, but those that did not achieve the LVEF recovery had a trend to earlier CV-specific mortality.

In CARDIOTOX, severe cardiotoxicity meant a 10-fold increase in the total mortality compared with mild or no CTRCD. Abdel-Qadir et al. published a study in which women with early-stage breast cancer died mostly of cancer, but those above 66 years with at least 5 years of survival had more mortality for CV causes than cancer (25). In our study, 30.9% of patients died, with only 22.8% of deaths related to the CV causes. In the patients with CTRCD, the most critical issue for the prognosis seemed not to be the cardiac problem, but also non-CV causes and cancer. Thus, the establishment of multidisciplinary cardio-oncology units can help avoid discontinuing the cancer treatment, as the prognosis is linked mostly to their cancer.

Cancer therapy-related cardiac dysfunction diagnosis is the most common indication for chemotherapy interruption (up to 38.1% in our cohort) (26). For instance, the patients with HER2-breast cancer with early trastuzumab interruption had higher rates of cancer recurrence and death than the patients receiving uninterrupted treatment (8). This fact could explain that trastuzumab was identified as a predictor of mortality in our cohort, emphasizing the negative impact of chemotherapy withdrawal on the prognosis.

Our study has some limitations. First, there were some missing visits or incomplete data collection during the follow-up related to the research nature of a retrospective registry. Second, all the CTRCD and LVEF recovery diagnoses were based only on LVEF, and we did not use other parameters, such as GLS (3). Also, we cannot have biomarkers (i.e., natriuretic peptides or Troponin) in most of the patients. Third, all the patients did not start cardiac-specific treatment due to the changing recommendations during the inclusion period. Finally, there was no specific follow-up protocol, and this meant that it was performed according to the usual clinical practice.

## Conclusions

In a real-world scenario, we have shown that up to 55% of the patients with CTRCD achieve LVEF recovery. Cardiac-specific treatment was given to 66% of the patients. The predictors of LVEF recovery were LVEF at CTRCD diagnosis, age, and time from starting the chemotherapy to cardiac dysfunction. Conversely, the predictors of mortality were trastuzumab treatment and LVEF at the time of CTRCD. Of note, only 23% of our patients died of CV causes. Our findings emphasize the need to develop cardio-oncology units to make an early diagnosis of CTRCD and initiate the cardiac treatment to improve the prognosis of the patients with cancer.

## Data Availability Statement

The raw data supporting the conclusions of this article will be made available by the authors, without undue reservation.

## Ethics Statement

The studies involving human participants were reviewed and approved by Clinica Universidad de Navarra, Pamplona, Spain. The Ethics Committee waived the requirement of written informed consent for participation.

## Author Contributions

AE-F and JG-C: conception and design or analysis, interpretation of data, drafting of the manuscript, revising it critically for important intellectual content, and final approval of the manuscript submitted. JC and JG-G: conception and design or analysis and final approval of the manuscript submitted. SP, AG, ÁS-G, and IF-R: drafting the manuscript or revising it critically for important intellectual content and final approval of the manuscript submitted. PM: interpretation of data and final approval of the manuscript submitted. All authors contributed to the article and approved the submitted version.

## Funding

Fundación para la Investigación Biomédica del hospital Universitario Puerta de Hierro supports authors with fees to open access.

## Conflict of Interest

SP has received a travel and accommodation grant from Novartis and advisor/consultant role for AstraZeneca, Daiichi-Sankyo, Polyphor, Roche, and Seattle Genetics. The remaining authors declare that the research was conducted in the absence of any commercial or financial relationships that could be construed as a potential conflict of interest.

## Publisher's Note

All claims expressed in this article are solely those of the authors and do not necessarily represent those of their affiliated organizations, or those of the publisher, the editors and the reviewers. Any product that may be evaluated in this article, or claim that may be made by its manufacturer, is not guaranteed or endorsed by the publisher.

## Abbreviations

CTRCD: cancer therapy-related cardiac dysfunction
CV: cardiovascular
ESC: European Society of Cardiology
HF: heart failure
LVSD: left ventricular systolic dysfunction
NYHA: New York Heart Association.
